# Discovery of Regulatory Elements is Improved by a Discriminatory Approach

**DOI:** 10.1371/journal.pcbi.1000562

**Published:** 2009-11-13

**Authors:** Eivind Valen, Albin Sandelin, Ole Winther, Anders Krogh

**Affiliations:** 1 The Bioinformatics Centre, Department of Biology and the Biotech Research and Innovation Centre (BRIC), University of Copenhagen, Copenhagen, Denmark; 2 DTU Informatics, Technical University of Denmark, Lyngby, Denmark; University of British Columbia, Canada

## Abstract

A major goal in post-genome biology is the complete mapping of the gene regulatory networks for every organism. Identification of regulatory elements is a prerequisite for realizing this ambitious goal. A common problem is finding regulatory patterns in promoters of a group of co-expressed genes, but contemporary methods are challenged by the size and diversity of regulatory regions in higher metazoans. Two key issues are the small amount of information contained in a pattern compared to the large promoter regions and the repetitive characteristics of genomic DNA, which both lead to “pattern drowning”. We present a new computational method for identifying transcription factor binding sites in promoters using a discriminatory approach with a large negative set encompassing a significant sample of the promoters from the relevant genome. The sequences are described by a probabilistic model and the most discriminatory motifs are identified by maximizing the probability of the sets given the motif model and prior probabilities of motif occurrences in both sets. Due to the large number of promoters in the negative set, an enhanced suffix array is used to improve speed and performance. Using our method, we demonstrate higher accuracy than the best of contemporary methods, high robustness when extending the length of the input sequences and a strong correlation between our objective function and the correct solution. Using a large background set of real promoters instead of a simplified model leads to higher discriminatory power and markedly reduces the need for repeat masking; a common pre-processing step for other pattern finders.

## Author Summary

In the years following the sequencing of the human genome focus have shifted towards trying to understand how this blueprint results in the diversity of cells that we observe. Part of the answer lies in the regulation of transcription and how the proteins responsible for this recognize where they should attach to the DNA. This is a well studied problem, but most methods developed for this have a hard time dealing with the heterogeneity of the mammalian genomes. Here we present a method that greatly improves the efficiency of this search by contrasting the DNA with a large number of background DNA sequences. This enables us to handle repetitive segments of the genome that may be functional, but are usually considered intractable by most methods.

## Introduction

The rapid emergence of experimental techniques that can probe for functional elements at whole-genome scales[1] necessitates computational methods to analyze data in these settings. In particular, methods that locate promoters or measure gene expression on genome-wide scales (e.g. [2],[3]) must be complemented by algorithms that can find the active regulatory elements within the larger promoters. *Ab initio* computational search for transcription factor binding sites (TFBS) in DNA sequences is often termed “motif discovery”. “Motif” here refers to a general pattern describing what DNA sequences the transcription factor binds[4]. Motif discovery is one of the classical problems in computational sequence analysis and can be briefly stated as: Given a set of sequences containing one or several short overrepresented sites, locate these and produce a model describing them.

There are two main avenues used to attack this problem: i) enumerative algorithms based on word counting, such as [5],[6], and ii) pattern-based approaches often using position specific weight matrices (WMs), which scores sites based on position specific weights [4]. Since the binding preferences of transcription factors (TFs) are not easily captured by a single word or consensus string, pattern-based approaches can give solutions closer to the biological reality and it has been argued that the matrix score is related to the binding energy [7],[8]. However, such approaches correspond to the problem of finding local, optimal multiple alignments, which is NP-complete [9]. Therefore, almost all pattern-based motif finders use statistical optimization methods such as Gibbs sampling or expectation maximization [10],[11].

A typical instance of motif discovery starts with a set of upstream promoter regions of co-expressed genes suspected to be co-regulated and by extension more likely to be under control by the same regulatory machinery. This set is called the “positive set” and most methods proceed from here by locating motifs that are in some way statistically overrepresented in this set. The most successful applications of motif discovery have been in organisms whose regulatory information is densely aggregated around transcription start sites, such as *Saccharomyces cerevisiae* (baker's yeast). In mammalian genomes, regulatory information is spread out over wider regions, which makes “pattern drowning” a significant issue; in other words, the information in the regulatory sites is too small to stand out in the large genomic region of interest. In this context, the accuracy of contemporary pattern finders is not sufficient for many biologically important problems [12].

Most methods operate with some notion of a background model describing “generic DNA” against which the over-representation is measured. The model is often a multinomial or a Markov model. The choice of model is important for obtaining good results [13],[14]. However, most such models have difficulty in capturing the complexity of the highly heterogeneous mammalian genome sequence, which has a multitude of different promoter architectures[15], numerous interspersed repeats, low complexity sequences, CpG islands, etc. [16]. Instead of simplifying the underlying DNA sequence by a general model, we take this to its extreme conclusion and use a very large set of promoters as the actual background instead of building a model describing the sequences in the promoters. For simplicity, we use the term “negative set” to describe the background set; this is strictly speaking not true as sites could occur in this set at a much lower frequency, since real promoters are sampled randomly. By contrasting the sets, it is possible to see what common features make the sequences in the positive set unique.

Discriminatory motif searching is not a new idea; several methods have been developed that take advantage of a negative set [17]–[24]. However, many of these use word-based models [19]–[21], which might not capture the diversity of binding sites. Others again use PWMs, but have binary hit models that do not distinguish between hits as long as they are over a threshold [22]. A discriminatory approach similar to ours has been combined with the use of expression data [18], but depending on the regions that are being investigated this might often not be available or even possible. We adopt an approach similar to DEME [23] to identify the most discriminative set of motifs by modeling the sequence labels (positive or negative) rather than using the conventional generative approach[10],[11]. However, there are some important differences to DEME. Firstly, DEME uses a global string-based search followed by a local gradient refinement, which may miss patterns that are not well-represented by a consensus string, whereas we use a global optimization technique (simulated annealing) for optimizing the model, which does not have this limitation, although it may have others (see below). Secondly, our method (Motif Annealer - MoAn) uses and optimizes a threshold, and uses an enhanced suffix array (ESA) to speed up pattern searches. Thirdly, in MoAn the length of the motif is also optimized. DEME is also particularly targeted towards proteins while our approach is intended for use with DNA.

Specifically, we use conditional maximum likelihood to estimate the WMs and their thresholds such that the probability of the positive and negative sets is maximized (see Methods). Thus, the resulting matrices cannot be derived from the frequency matrix for the sites found – it is rather the matrices that lead to the best discrimination. The probability of a sequence is calculated as a product of the probabilities given by the matrices matching above a threshold and a simple null model for non-matching regions. From this and prior probabilities for matches in the positive and negative sets, the probability of the set label (positive or negative) is calculated. In this probability the background model cancels. The total likelihood is a product of the class probabilities for all sequences (positive and negative).

This conditional likelihood leads to a non-trivial optimization problem which is handled using simulated annealing (see Methods), where we iteratively change the WMs and their thresholds, retaining changes that lead to higher discriminatory power using the Metropolis-Hastings algorithm [25],[26]. Given sufficient iterations, the method guarantees convergence on the optimally discriminatory motifs. To cope with the vast size of the sets we utilize a highly efficient data structure, the ESA, for searching DNA for pattern instances[27]. With reasonable cutoffs, this reduces the computation by an order of magnitude[28].

## Results

We evaluated our method by comparing its accuracy to a set of widely used motif discovery methods (MEME[29], DEME[23], Weeder[5] and NestedMICA[14]) in several different ways. In all runs, we used the same background set, which consists of 1000 experimentally defined promoters randomly sampled from the mouse genome (Text S1). The evaluation statistics are the same as used in [12] (see Methods) and we also pooled the results from all motifs (grouped by length of the input sequence; see below) and calculated the compound statistics on this. To reduce the influence of the optimization method, we ran all non-deterministic methods five times on each set selecting the best run according to their own scoring function.

In line with the recommendations of [12] we used synthetic data sets for the inter-method comparison. These were constructed by taking experimentally defined promoter regions based on strong CAGE tag clusters [2] and planting binding sites from various TFs inside these (Text S1). To decrease possible biases for the methods towards certain specific motif types, we randomly selected one TF from each of the 11 JASPAR[30] families as well as an example of a zinc-finger factor (Table S1). For a given matrix, we randomly chose sites from experimentally validated binding sequences used for constructing the JASPAR matrix instead of generating sites using the matrix. Since the accuracy of motif discovery methods normally deteriorates when sequence length is increased (“pattern drowning”), we evaluated the various methods on sets with sequence lengths varying between 200 and 1200 nucleotides (Table S3). This gave a total of 84 sets (12 motifs ×7 lengths) with 100 sequences in each. Sequences had a site from a given motif planted with a probability of 0.5. For those methods that support it, a background/negative set was provided containing 1000 sequences sampled in the same way and with the same length as the positive sequences. We used default settings for all methods except where there were obvious reasons not to (Text S2). Since DEME requires motif length as input we decided to input the correct length of the matrix. This provides DEME with an informational advantage over the other methods.

**Figure 1:**
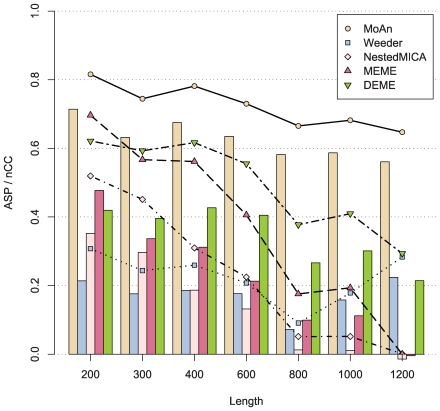
Synthetic set evaluation. The average site performance (lines) and the nucleotide correlation coefficient (bars) of the methods.

Fig. 1 (and Figs. S4, S5, S6, S7, S8) shows a significant performance gain in using MoAn compared to the other methods as measured by Matthews correlation coefficient on nucleotide level (nCC) and average site performance (ASP) – an average over the positive predictive value and the sensitivity on binding site level (see Methods for details). With both measures, MoAn performs better than any other method on all sequence lengths. In particular, the performance is not as affected by increasing the input sequence length as the other methods; at certain sequence lengths(800, 1200) MoAn has more than twice as high ASP values as the second best method. We also evaluated MoAn with the applicable subset of the evaluation set proposed by [12](Text S3 and Table S4), where the OligoDyad, AnnSpec and MoAn achieve the highest sASP values. We note that this set is challenging as none of the methods perform well overall, and the difference in performance between methods might not be significant due to this fact. In addition, this set does not evaluate how well the method can deal with increasing lengths of input sequences, which is highly relevant.

### Correlation of score and solution

**Figure 2:**
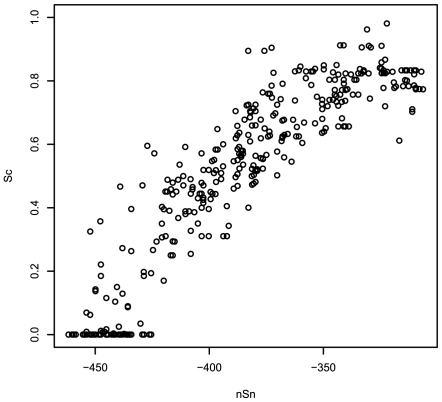
Correlation of MoAn's objective function (Sc) and site sensitivity (sSn). All 5 runs on the 84 synthetic sets are used.

The relationship between our objective function and the correct solution was assessed by plotting the MoAn scores against the sensitivity obtained in all five runs on each of the 84 sets (not just the best from each run) (Fig. 2). There is a clear correlation (Pearson CC: 0.90) between these two measures. There is a similar correlation with other measures, such as the nCC (Fig. S1).

This finding is important, because it indicates that the raw score is an indication of quality independent of the motif analyzed. It also shows that choosing the best scoring run of several will often give the best result.

### Repetitive sequences

**Figure 3:**
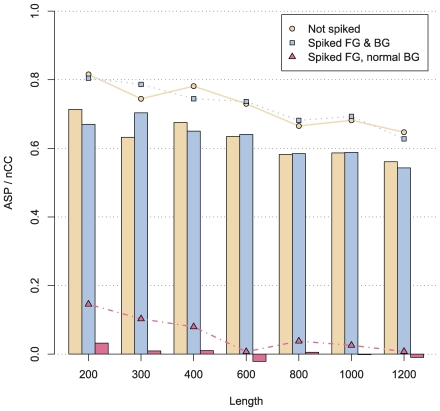
Repeat Assessment. The average site performance (lines) and the nucleotide correlation coefficient (bars) of MoAn with repeats planted in the two sets.

Aside from the problem with decreasing sensitivity as the length of the input sequences increase, repetitive sequences represent a severe problem for motif discovery, as these will often seem to be over-represented, and therefore it is common to mask these repeats. However, masking is always arbitrary, and some repeats are functional [31],[32], so indiscriminate repeat masking is not optimal. When using a large negative set, repeat masking is unnecessary since repeats, if commonly occurring, will feature in the negative set and therefore be avoided as potential hits in the positive. At the same time, we can avoid the reverse problem – if a type of repeat actually is over-represented in the positive set, it can still be found. To demonstrate the insensitivity to repeats on a practical level, we planted repetitive sequences in each of the positive sets with a slightly higher frequency than the real motifs and ran our predictor on these sets both with the normal background and with a background similarly spiked with repeats. Specifically, we planted 1 to 10 consecutive instances of CACTA with a probability of 60% in each sequence. Fig. 3 shows, as expected, that the results do not deviate much from the repeat-less run when repeats are planted in both the positive and negative sequences, while the method picks up the repeats instead when there are no repeats in the negative set. We also performed this test using decoy motifs instead of repeats with similar results (Text S4, Fig. S2).

### Real data

Evaluation of methods on real data is difficult and often a poor indication of general performance due to lack of insight into the correct solution [12]; on the other hand, it is necessary to show that the method can be applied to real problems.

MoAn and four other methods were run on a collection of real data sets consisting of the binding sites of four human and mouse factors from the PAZAR database[33] and their associated genomic sequence. The sets were split by organism into 7 sets and the regions adjacent on the genome were merged resulting in sets ranging in size from 14 to 118. The merging means that the base sequences can have a varying number of sites and may be of different lengths. The sets were then subsequently enlarged by adding an equal number of randomly selected promoters to increase the difficulty (Text S6 and Table S5) and also padded with their cognate upstream and downstream regions of varying lengths (200–1200, as in the synthetic evaluation) to estimate the impact of noise.

**Figure 4:**
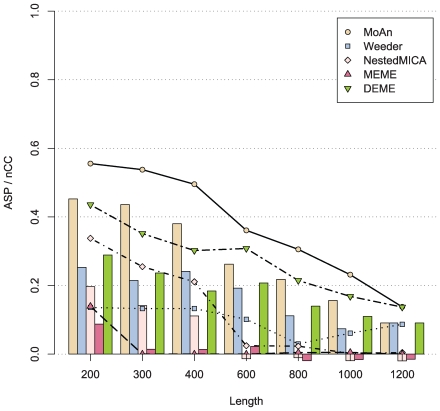
PAZAR set evaluation. The average site performance (lines) and the nucleotide correlation coefficient (bars) of the methods.

Fig. 4 shows the performance over the real sets. MoAn's performance is clearly superior, but not as spectacular as in the more controlled environment with synthetic sequences.

We speculate that the reason for this is that the background and foreground of the synthetic sets are essentially sampled from the same pool (RefSeq promoters), while we have made no effort to customize the background for the PAZAR sets. If the genomic environment of the factors differ from normal promoter sequences this could lead to a reduced performance. There are also fewer sets (7 versus 12) in this evaluation leading to a higher variability.

We report additional trials using ChIP-chip data in supplementary material (Text S7, Fig. S3 and Tables S6, S7). MoAn has also been used successfully to discriminate between binding regions of human ESR1 and its paralog ESR2; the results were comparable with matrix-scanning approaches with pre-defined motifs[34].

### Co-occurrence of binding sites

**Figure 5:**
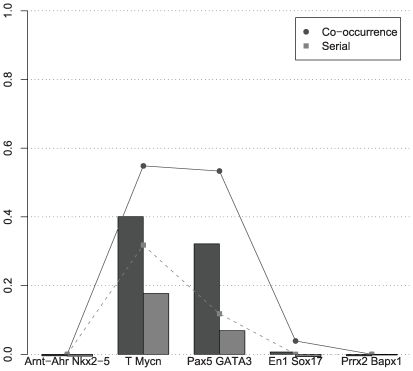
Performance of co-occurrence vs. serial runs. The average site performance (lines) and nucleotide correlation coefficient (bars) of co-occurrence and serial runs on 5 different sets with co-occurring motifs.

An additional aspect of the motif finding problem is that TFs often work by forming complex interactions [35]. Examples include mutually exclusive and cooperative binding. Clusters of TFBSs are commonly termed cis-regulatory modules, and are often responsible for tissue-specific expression. We try to capture these interactions by incorporating co-occurrence of sites from different motifs into our model, with the goal of further increasing predictive power. To test whether our objective function is capable of capturing interactions between factors we constructed a set where co-occurrence of sites from different motifs occurs. We randomly chose 5 pairs of new motifs (Table S2) and planted their corresponding sites in a positive set of 100 promoters with a 40% chance of co-occurrence and 10% of single occurrence. We then spiked the background set with sites from each of the motifs (10% chance each for all sequences) to mimic a situation where it is the interactions of the two sites rather than single sites that are responsible for the regulation. MoAn was then run in co-occurrence mode and compared to two single-occurrence runs in a series. In the serial runs we masked out the predictions from the first iteration before running the second iteration. In Fig. 5 the ASP and nCC is plotted. In our experiment three of the pairs turned out to be composed of motifs with relatively low information, leading to poor performance. However, the two remaining ones show that modeling of co-occurrence can significantly improve performance. This extended model is unfortunately computationally taxing and requires more than twice the number of iterations compared to the single prediction.

## Discussion

In this work we have shown the value of using a large negative set instead of a pre-defined background model in motif discovery. Using raw sequences more accurately portrays the background than any general model and therefore higher discriminatory power is achieved. This method is also much less sensitive to “pattern drowning” in larger sequences, which is a bottleneck in computational analysis of mammalian regulatory regions. However, while our method takes a significant step towards routine motif discovery on large sequences, the problem cannot be considered fully solved. In particular, MoAn accuracy may be further improved by incorporating information on evolutionary constraints (phylogenetic footprinting)[36] or DNA accessibility[24],[37].

In our opinion DEME is the best runner up of the methods. It often predicts the correct motif and has a high sensitivity, but often at the cost of a large number of false positives as it predicts also in those sequences not containing a site. MoAn seems to be better at balancing the sensitivity and specificity. On the other hand DEME is also given an artificial advantage by having the correct motif length as input and it is uncertain how advantageous this is.

Weeder performed surprisingly poorly given its stellar performance in a recent evaluation[12]. This might be due to motif selection which we did according to the most redundant motif, but was in [12] done in a more complicated manner not part of the current Weeder package. This procedure led to no predictions on several of the harder sets which might give Weeder a statistical advantage (as discussed in [12]).

A concern that might be raised is that optimizing a cutoff might lead to a conservative estimate of binding sites at the expense of weaker sites. However, assessing this is hard since experiments have their own thresholds in the post-analysis and any evaluation of MoAn's threshold will be dependant upon those. Investigations where we artificially forced the cutoff to remain low, lead to a reduction in performance (data not shown). We address this potential problem indirectly by providing a matrix that can be used to search sequences at a lower threshold.

Future improvements of MoAn will focus on the optimization algorithm, which currently is not robust enough to always produce reliable results. In our current implementation we avoid this problem by running the algorithm many times to see that the solution is stable.

## Methods

Evaluation is done on both site and nucleotide levels. The statistics used are similar to those in the recent large scale evaluation [12]. To get a compound statistic for all motifs at each length we used what is there described as the “combined” method for summarizing. This consists of treating all sets of a given length as one big set, summing up all the basic statistics below (nTP, nTN … sFN) before calculating the compound statistics. This removes the problem of undefined statistics in those cases where a method does not predict any sites.

### Basic statistics

nTP Number of nts part of a site correctly predicted.

nTN Number of background nts correctly predicted.

nFP Number of background nts predicted to be part of a motif.

nFN Number of nts part of a site predicted as background.

sTP Number of real sites that share over 50% of its nts with a predicted site.

sFP Number of predicted sites that share less than 50% of its nts with a real site.

sFN Number of real sites that share less than 50% of its nts with a predicted site.

Note that we are more conservative with respect to the site prediction than [12] in that we demand at least half of the nucleotides overlapped to get a single sTP.

### Compound statistics

Derived from the basic statistics:
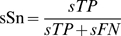


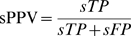


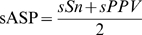


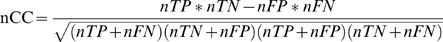



### Objective function

A sequence 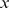 is assumed to be described by a mixture model consisting of a background distribution 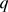 and a set of WMs 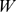 describing the binding affinities of the TFs. The WMs contain log-odds scores of the type:
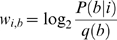
(1)where 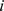 is the position in the WM, 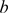 is a letter in the DNA alphabet and 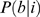 is the probability of having letter 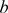 at position 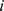 in the motif described by 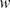. The score of a matrix 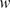 aligned at a position 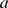 in a sequence 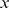 is therefore:
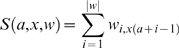
(2)where 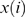 is the DNA letter at position 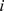 in sequence 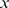.

The aim is to discriminate between two sets of sequences 
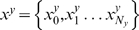
, where label 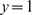 denotes the positive set and 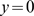 the negative. The prior probability of binding site occurrence in a sequence contained in set 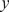 is called 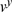. We assume that there is a marked difference in the site occurrence between the two sets and want to construct a score that captures how well a set of WMs describe this difference. Using two WMs as an example, 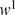 and 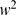, there are four possible ways for a sequence 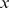 to be generated. With prior probability 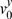 it contains no sites and is only generated by the background model 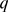. Or, with prior probability 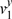, it contains a single site (one of the two) corresponding to one WM 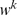 positioned at nucleotide number 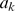 (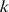 is equal to 1 or 2 corresponding to the two different matrices). This is written 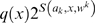, where 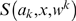 is the score of the matrix aligned to the nucleotides at position 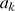 (eq. 2) and 2 is the base of the log scores contained in the WM. Note that the log scores in a WM are divided by the background model, so the background (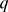) cancels out in sites where the motif occurs. The final case, with prior probability 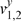, is the co-occurrence of two sites in a sequence, which is 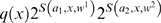. However, this is only correct when the sites are not overlapping since otherwise the overlapping nucleotides would be included in the product twice. Therefore we disallow overlaps.

For efficiency reasons, we do not calculate the score in its entirety. We assume that it is the strong sites that contribute the most to the equation and introduce a cutoff for each WM on the minimum score of a site. This enables an efficient search in the ESA. This is not without biological merit since WM scores and binding energies for known TFs are correlated, and at some point the binding energies of a TF and a poor binding sequence must be too small to matter [4]. It is also a standard method to use when scanning with known matrices [38]. So we only consider sites that score above a threshold, which is called 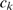 for matrix 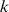. Then the probability of a sequence 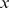 from the set 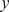 being generated by the WMs is
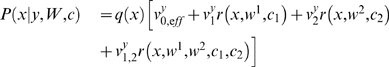
(3)where 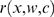 is the expectation over 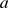 of 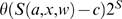 over all predicted sites:
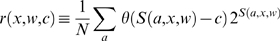
(4)with 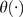 being the step function (1 above 0 and zero otherwise). The co-occurrence expectation 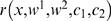 is defined in a similar way with overlaps disallowed. The effective weight of no sites
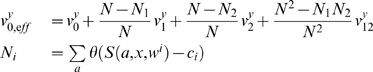
(5)accounts for extra weight given to no sites due to alignments not meeting the threshold. With this definition, 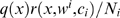 is the probability or generative model of the sequence conditioned on the WM and threshold, 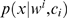.

To find the WMs that best explain the difference in occurrence between the sets we use a discriminative objective function based on the probability of the labels 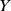 given the sequences 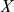 and WMs, formally:
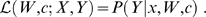
(6)


This is the logistic likelihood function for binary classification, see e.g. [39]. The discriminative model can thus be viewed as logistic regression with an adaptive set of basis functions. For multiple sequences assumed to be independent, the joint probability is the product of the single sequence probabilities over all sequences in both the positive and negative set:
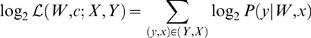
(7)


We refer to this function as the (log likelihood) score, 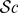.

Based on the sequence density 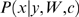 we can use Bayes theorem to calculate the probability of the label 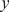 given the WMs 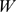, the thresholds 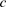, and the sequence 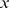:
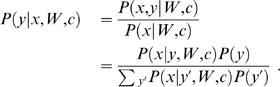
(8)


We observe that the prior probability of 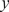 is proportional to the number of sequences in the set divided by the total number of sequences 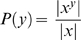.

A very high threshold will give no matches, and the probability will then be a constant given by the priors and the size of the two sets. Matches that score above the threshold in the negative set will lower the score and matches above the threshold in the positive set will increase the score, so the game is to obtain as many high-scoring matches in the positive set as possible without introducing too many matches in the negative set.

The prior is conservative in our runs in that we are strict about promoting hits in the positive set, but only moderately strict about disallowing negative hits. For a single matrix the prior on 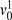 is 0.01; 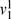: 0.99; 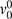: 0.80; 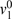: 0.20. For two matrices: 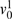: 0; 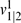: 0.1; 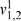: 0.9; 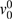: 0.80; 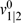: 0.15; and 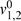: 0.05. These priors can be set by the user if prior knowledge is available about the set (i.e. a high confidence negative set or an uncertain positive set).

In the evaluation we deliberately chose a probability of having a site (0.5) in a sequence very different from the model prior (0.99) to avoid giving our own method a big advantage. It shows that the method is not very sensitive to the choice of prior.

### Optimization

The objective function outlined above is optimized using simulated annealing [40]. Informally, it proceeds by iteratively proposing a candidate solution and then accepting or rejecting it depending on how good it is compared to the current solution. It sometimes accepts changes for the worse and therefore possesses the power to escape local maxima. The hope is that it will converge on a solution that is close to optimal. Formally, this translates to a walk over the search space 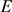 where in the current state 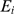, the next state 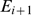 is either the same or the candidate solution 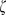 depending on their relative scores and a temperature parameter 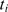.
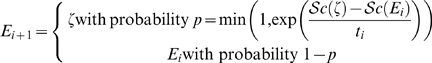



The temperature parameter is lowered for each iteration using as default an exponential cooling scheme (for details see Text S5), thus incrementally constraining the neighborhood of accepted changes.

Candidate solutions are proposed by applying one of several steps outlined in the list below. In the case of multiple matrices, only one is changed at a time. We perform all steps on a integer “count” matrix which is then translated into a log-odds WM prior to searching the ESA, but notice that the “count” matrix does not represent actual letter frequencies in the selected sites. The steps are:

- Alter the contents of the WM columns by moving counts from one random cell to another within a column. The number of counts moved is selected uniformly from 1 to the current count number for the cell.
- Extend the WM in either direction. A uniformly sampled number of columns (1 to 5) is added and counts of these are decided by consulting the sequence locations of hits scoring above 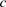. The counts are proportional to the counts in the columns from the extended hits, but normalized so that all columns have the same counts.
- Decrease the length of the WM by deleting columns. Similarly to adding columns a uniformly selected number between 1 and 5 columns are deleted.
- Slide the WM across the sequences. Columns are deleted on one site and extended on the other according to the two steps above.
- Alter the cutoff 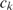 of the matrix 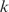. The cutoff is expressed in bits per column and a new candidate 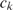 is proposed by sampling uniformly from 0.6 to 2 bits.

Note that for the extend and decrease step there is a minimum and maximum number of columns for a motif. The default for these are 5 and 15 respectively.

The matrix is initialized with random counts and the cutoff is also selected uniformly according to the last step in the list above. Termination of the optimization is only based on the number of iterations which is by default set to a rather conservative value of 30 million iterations. Time requirements for a single run is variable depending on the set size, but was for our runs comparable to NestedMICA (single threaded) and considerably faster than Weeder's “large” run and DEME.

### Availability

Source code as well as data sets is freely available at the author's web site: http://moan.binf.ku.dk

## Supporting Information

Figure S1 Correlation of MoAn's objective function (Sc) and nucleotide correlation coefficient (nCC)
(0.01 MB EPS)

Figure S2 Evaluation with decoy motifs. Average site performance (lines) and the nucleotide correlation coefficient (bars) of MoAn with decoy motifs planted in the two sets.
(0.01 MB EPS)

Figure S3 Discriminatory power of matrices. ROC curve showing discriminatory power of matrices produced by MoAn and NestedMICA on the ESR1 data set. The line extends from the highest cutoff possible for that matrix (bottom right) to a cutoff of 0 (top left).
(0.03 MB EPS)

Figure S4 Performance on individual sets for MoAn. The average site performance (lines) and the nucleotide correlation coefficient (bars) on the sets.
(0.02 MB EPS)

Figure S5 Performance on individual sets for DEME. The average site performance (lines) and the nucleotide correlation coefficient (bars) on the sets.
(0.02 MB EPS)

Figure S6 Performance on individual sets for MEME. The average site performance (lines) and the nucleotide correlation coefficient (bars) on the sets.
(0.02 MB EPS)

Figure S7 Performance on individual sets for Weeder. The average site performance (lines) and the nucleotide correlation coefficient (bars) on the sets.
(0.02 MB EPS)

Figure S8 Performance on individual sets for NestedMICA. The average site performance (lines) and the nucleotide correlation coefficient (bars) on the sets.
(0.02 MB EPS)

Text S1 Data set construction
(0.03 MB PDF)

Text S2 Running parameters
(0.03 MB PDF)

Text S3 Tompa assessment
(0.03 MB PDF)

Text S4 Sequences spiked with decoy motifs
(0.02 MB PDF)

Text S5 Annealing schedule
(0.03 MB PDF)

Text S6 PAZAR data sets
(0.03 MB PDF)

Text S7 ChIP-chip data sets
(0.04 MB PDF)

Table S1 Length of upstream and downstream extensions
(0.01 MB PDF)

Table S2 Motifs planted in single occurrence sets
(0.04 MB PDF)

Table S3 Motifs planted in co-occurrence sets
(0.03 MB PDF)

Table S4 Results on the mammalian subset of the Tompa assessment
(0.01 MB PDF)

Table S5 Sizes of PAZAR data sets
(0.01 MB PDF)

Table S6 Sizes of ENCODE data sets
(0.01 MB PDF)

Table S7 Performance on ENCODE data sets
(0.07 MB PDF)

## References

[1] Birney E, Stamatoyannopoulos J, Dutta A, Guigó R, Gingeras T (2007). Identification and analysis of functional elements in 1% of the human genome by the ENCODE pilot project.. Nature.

[2] Carninci P, Kasukawa T, Katayama S, Gough J, Frith MC (2005). The transcriptional landscape of the mammalian genome.. Science.

[3] Kim T, Barrera L, Zheng M, Qu C, Singer M (2005). A high-resolution map of active promoters in the human genome.. Nature.

[4] Stormo GD (2000). DNA binding sites: representation and discovery.. Bioinformatics.

[5] Pavesi G, Mauri G, Pesole G (2001). An algorithm for finding signals of unknown length in DNA sequences.. Bioinformatics.

[6] Sinha S, Tompa M (2003). YMF: A program for discovery of novel transcription factor binding sites by statistical overrepresentation.. Nucleic Acids Res.

[7] Stormo GD (1998). Information content and free energy in DNA-protein interactions.. J Theor Biol.

[8] Berg G, von Hippel P (1987). Selection of DNA binding sites by regulatory proteins.. J Mol Biol.

[9] Wang L, Jiang T (1994). On the complexity of multiple sequence alignment.. J Comput Biol.

[10] Cardon LR, Stormo GD (1992). Expectation maximization algorithm for identifying protein-binding sites with variable lengths from unaligned DNA fragments.. J Mol Biol.

[11] Lawrence CE, Altschul SF, Boguski MS, Liu JS, Neuwald AF (1993). Detecting subtle sequence signals: a Gibbs sampling strategy for multiple alignment.. Science.

[12] Tompa M, Li N, Bailey TL, Church GM, De Moor B (2005). Assessing computational tools for the discovery of transcription factor binding sites.. Nat Biotechnol.

[13] Thijs G, Lescot M, Marchal K, Rombauts S, De Moor B (2001). A higher-order background model improves the detection of promoter regulatory elements by Gibbs sampling.. Bioinformatics.

[14] Down TA, Hubbard TJ (2005). NestedMICA: sensitive inference of over-represented motifs in nucleic acid sequence.. Nucleic Acids Res.

[15] Carninci P, Sandelin A, Lenhard B, Katayama S, Shimokawa K (2006). Genome-wide analysis of mammalian promoter architecture and evolution.. Nat Genet.

[16] Lander ES, Linton LM, Birren B, Nusbaum C, Zody MC (2001). Initial sequencing and analysis of the human genome.. Nature.

[17] Workman C, Stormo G (2000). ANN-Spec: a method for discovering transcription factor binding sites with improved specificity..

[18] Segal E, Barash Y, Simon I, Friedman N, Koller D (2002). From promoter sequence to expression: a probabilistic framework..

[19] Sinha S (2003). Discriminative motifs.. Journal of Computational Biology.

[20] Takusagawa K, Gifford D (2004). Negative information for motif discovery..

[21] Sumazin P, Chen G, Hata N, Smith A, Zhang T (2005). DWE: discriminating word enumerator.. Bioinformatics.

[22] Leung H, Chin F (2006). Finding motifs from all sequences with and without binding sites.. Bioinformatics.

[23] Redhead E, Bailey T (2007). Discriminative motif discovery in DNA and protein sequences using the DEME algorithm.. BMC bioinformatics.

[24] Narlikar L, Gordân R, Hartemink A, Miyano S (2007). A nucleosome-guided map of transcription factor binding sites in yeast.. PLoS Comput Biol.

[25] Metropolis N, Rosenbluth A, Rosenbluth M, Teller A, Teller E (1953). Equation of state calculations by fast computing machines.. The journal of chemical physics.

[26] Hastings W (1970). Monte Carlo sampling methods using Markov chains and their applications.. Biometrika.

[27] Beckstette M, Homann R, Giegerich R, Kurtz S (2006). Fast index based algorithms and software for matching position specific scoring matrices.. BMC bioinformatics.

[28] Marstrand TT, Frellsen J, Moltke I, Thiim M, Valen E (2008). Asap: a framework for over-representation statistics for transcription factor binding sites.. PLoS ONE.

[29] Bailey TL, Elkan C (1994). Fitting a mixture model by expectation maximization to discover motifs in biopolymers.. Proc Int Conf Intell Syst Mol Biol.

[30] Bryne JC, Valen E, Tang MH, Marstrand T, Winther O (2008). JASPAR, the open access database of transcription factor-binding profiles: new content and tools in the 2008 update.. Nucleic Acids Res.

[31] Romanish MT, Lock WM, van de Lagemaat LN, Dunn CA, Mager DL (2007). Repeated recruitment of LTR retrotransposons as promoters by the anti-apoptotic locus NAIP during mammalian evolution.. PLoS Genet.

[32] Buzdin A, Kovalskaya-Alexandrova E, Gogvadze E, Sverdlov E (2006). GREM, a technique for genome-wide isolation and quantitative analysis of promoter active repeats.. Nucleic Acids Res.

[33] Portales-Casamar E, Kirov S, Lim J, Lithwick S, Swanson M (2007). PAZAR: a framework for collection and dissemination of cis-regulatory sequence annotation.. Genome Biology.

[34] Liu Y, Gao H, Marstrand TT, Strom A, Valen E (2008). The genome landscape of ERalpha- and ERbeta-binding DNA regions.. Proc Natl Acad Sci U S A.

[35] Wasserman WW, Fickett JW (1998). Identification of regulatory regions which confer muscle-specific gene expression.. J Mol Biol.

[36] Lenhard B, Sandelin A, Mendoza L, Engstrom P, Jareborg N (2003). Identification of conserved regulatory elements by comparative genome analysis.. J Biol.

[37] Mellor J (2006). Dynamic nucleosomes and gene transcription.. Trends in Genetics.

[38] Wasserman WW, Sandelin A (2004). Applied bioinformatics for the identification of regulatory elements.. Nat Rev Genet.

[39] Bishop C (2006). Pattern recognition and machine learning. Springer New York..

[40] Kirkpatrick S, Gelatt J C D, Vecchi MP (1983). Optimization by simulated annealing.. Science.

