# Supplementary material for: Discovery of Regulatory Elements is Improved by a Discriminatory Approach
Source: PLoS Comput Biol. 2009 Nov 13;5(11):e1000562. doi: 10.1371/journal.pcbi.1000562 (PMC2770120; doi:10.1371/journal.pcbi.1000562)
Supplement: Text S1 — Data set construction (0.03 MB PDF) [file pcbi.1000562.s009.pdf]

# Supplementary Text S1: Data set construction

Data was constructed sampling strong tag clusters (TC) from [1]. TC is a concept introduced with the Cap Analysis of Genome Expression (CAGE) technology and is defined in the same paper. It correspond roughly to the traditional concept of a core promoter, but extended to a multiple TSS framework. TCs were eligible for sampling if they contained more than 30 CAGE tags from all CAGE libraries after each CAGE library has been normalized to a total of 1 million tags. This is traditionally considered a strong promoter.

The sequences used consists of the whole TC (the sequence containing all TSSs in the cluster) plus flanking regions of the appropriate length. These regions are not symmetric, but follow the distribution in table S1. The asymmetry is primarily to avoid including too much of the coding regions.

Note that the sampling is only performed once for each motif and then the length of the flanking regions around this set of TCs is varied. The procedure is the same for the positive and the negative set, but in the negative set we made sure that we did not sample any of the sequences already present in any of the positive sets.

## Planting sites and repeats

Sites were planted using the actual binding sites downloaded from the JASPAR[2] database with a probability of 50% in each sequence. The specific site was sampled uniformly from the set of sites used to construct the JASPAR matrix and the position uniformly from the whole sequence. Transcription factor JASPAR matrices for the single occurrence evaluation sets are listed in table S2.

The co-occurrence set was constructed similarly to the single occurrence. Transcription factor JASPAR matrices for the co-occurrence evaluation sets are listed in table S3.

The repeat set is the same as the original spiked set for (single occurrence) inter-method comparison, but with each sequence having a 60% chance of containing a repeat. Repeats were constructed by concatenating the string “CACTA” a random number of times between 1 and 10 which then replaced a sequence of equal length in the original sequence. We took care not to replace any planted motif site that the sequence might contain.

# References


[1] Carninci, P. et al. (2006) Genome-wide analysis of mammalian promoter architecture and evolution *Nat Genet* 38:626-635.

[2] Bryne, J.C. et al. (2007) JASPAR, the open access database of transcription factor-binding profiles: new content and tools in the 2008 update. *Nucleic Acids Res* 36:D102-D106.
