# Supplementary material for: Discovery of Regulatory Elements is Improved by a Discriminatory Approach
Source: PLoS Comput Biol. 2009 Nov 13;5(11):e1000562. doi: 10.1371/journal.pcbi.1000562 (PMC2770120; doi:10.1371/journal.pcbi.1000562)
Supplement: Text S2 — Running parameters (0.03 MB PDF) [file pcbi.1000562.s010.pdf]

Supplementary Text S2: Running parameters

MoAn

MoAn (v1.0) was run with the following command line:

```
moan POSITIVE NEGATIVE
```

For co-occurrence runs we also included "-c" denoting use of two matrices and for the real data we used "-D" denoting double stranded search.

DEME

DEME (v1.0) was run with the following command line:

```
deme -w LEN -p POSITIVE -n NEGATIVE
```

For the real data we included the switch "-revcomp" to use both strands. The LEN indicates the correct length of the motif as specified by the JASPAR matrix. Therefore, DEME enjoys an informational advantage over the other methods. In the case of rara that has no JASPAR matrix we based the length on other nuclear receptors.

MEME

MEME (v3.5.3) was run with the following command line:

```
meme -text -bfile meme.mm.bg4.1000 -dna POSITIVE
```

where the background file is a fourth order model constructed from the negative set with the fasta-get-markov tool that comes with MEME:

```
fasta-get-markov -m 4 < NEGATIVE > meme.mm.bg4.1000
```

For real data we included the switch "-revcomp" to use both strands and "-maxsize 1000000" to reserve enough memory to process the whole set.

Weeder

Weeder (v1.3) was run with the following command line:

```
weederlauncher.out POSITIVE MM large
```

The "S" option was included on the real data to include both strands and "HS" was used instead of "MM" for the ENCODE sets.

NestedMICA

NestedMICA (v0.7.3) was run with the following command line:

```
motiffinder -seqs POSITIVE -backgroundModel nmica.C4.O1.bg
```

Where the background model is a first order, four class model as suggested in the NestedMICA manual (http://www.sanger.ac.uk/Software/analysis/nmica/nmica-manual-0.7.3.pdf):

```
makemosaicbg -seqs NEGATIVE -mosaicClasses 4 -mosaicOrder 1 -out nmica.C4.O1.bg
```

Since NestedMICA finds a motif matrix, not a set of sites we needed to scan this across the sequences to obtain a prediction that could be used. This was done using:

```
motifscanner -seqs POSITIVE -motifs motifs.xms -scoreThreshold -5.0
```

The score threshold is chosen according to the typical usage example in the NestedMICA manual.
