# Supplementary material for: Discovery of Regulatory Elements is Improved by a Discriminatory Approach
Source: PLoS Comput Biol. 2009 Nov 13;5(11):e1000562. doi: 10.1371/journal.pcbi.1000562 (PMC2770120; doi:10.1371/journal.pcbi.1000562)
Supplement: Text S3 — Tompa assessment (0.03 MB PDF) [file pcbi.1000562.s011.pdf]

Supplementary Text S3: Tompa evaluation

This section and table S4 features the results on a subset of the Tompa evaluation[1]. This includes real and generic sequences for mouse and human. Markov were not included since we could not create a proper background model for this without knowing the parameters of the model and the two other organism were excluded since MoAn is geared towards mammalian motif finding. Unfortunately this is not a particularly good set for evaluation since it features too many unrealistically hard sets. Of the 26 sets that remains after filtering, 4 has only 2 sequences and as many as 14 of them has 5 sequences or less. To illustrate the difficulty one can note that none of the predictors, including MoAn, perform reliably on more than two or three sets. And that the highest nCC obtained is 0.069 (oligodyad) followed by MoAn at 0.066. It is doubtful how statistically significant these results are.

References

[1] Tompa, M. et al. (2005) Assessing computational tools for the discovery of transcription factor binding sites *Nat Biotechnol* 23:137-144.
