# Supplementary material for: Discovery of Regulatory Elements is Improved by a Discriminatory Approach
Source: PLoS Comput Biol. 2009 Nov 13;5(11):e1000562. doi: 10.1371/journal.pcbi.1000562 (PMC2770120; doi:10.1371/journal.pcbi.1000562)
Supplement: Text S4 — Sequences spiked with decoy motifs (0.02 MB PDF) [file pcbi.1000562.s012.pdf]

Supplementary Text S4: Sequences spiked with decoy motifs

This run (Figure S2) is similar to the repeat spiked run in that we spiked the sequences with a decoy motifs. However in this run we used PSSMs rather than repeat sequences. The PSSMs used in the single occurrence runs was paired randomly with another. We made two copies of the background sequences and spiked one of them with the real motif (0.5 probability) and the other with both the real and the decoy (with prob. of real: 0.5 and decoy: 0.6). The foreground sequences were also spiked with both motifs (same probabilities). The results (Fig. S2) are similar to the repeat spike experiment, but unlike the repeat experiment the decoy is not completely dominant here. This is probably due to different information content in the matrices. So that in the cases where the real is much more information rich than the decoy, the real gets picked up despite having a lower probability of appearing in each sequence.
