# Supplementary material for: Discovery of Regulatory Elements is Improved by a Discriminatory Approach
Source: PLoS Comput Biol. 2009 Nov 13;5(11):e1000562. doi: 10.1371/journal.pcbi.1000562 (PMC2770120; doi:10.1371/journal.pcbi.1000562)
Supplement: Text S5 — Annealing schedule (0.03 MB PDF) [file pcbi.1000562.s013.pdf]

Supplementary Text S5: Annealing schedule

We use an exponential cooling scheme where:

$$t_{i+1} = \alpha t_i \quad (1)$$

alpha is calculated based on the number of iterations:

$$\alpha = \exp \frac{\log(t_N/t_0)}{N} \quad (2)$$

Where N is the number of iterations. A linear cooling scheme can also be choosen through command line options, though we experienced lower performance using this in our runs.
