# Supplementary material for: Discovery of Regulatory Elements is Improved by a Discriminatory Approach
Source: PLoS Comput Biol. 2009 Nov 13;5(11):e1000562. doi: 10.1371/journal.pcbi.1000562 (PMC2770120; doi:10.1371/journal.pcbi.1000562)
Supplement: Text S6 — PAZAR data sets (0.03 MB PDF) [file pcbi.1000562.s014.pdf]

Supplementary Text S6: PAZAR data sets

Four PAZAR sets were downloaded from the PAZAR website[1] and divided into mouse and human sets (table S5). The other organisms were discarded. This resulted in 7 sets of varying sizes (table S5). Binding sites closer than 1200bp (potentially overlapping) were merged and treated as one sequence for extension purposes. The sets were then padded with an equal number of randomly sampled promoter sequences from the Ensembl[2] database presumably not containing the motif. The sets were then extended with their cognate genomic sequences on each side according to table S1.

References

[1] http://www.pazar.info

[2] Hubbard, TJP et al. (2009) Ensembl 2009 *Nucleic Acids Research* 37 D690
