# Supplementary material for: Discovery of Regulatory Elements is Improved by a Discriminatory Approach
Source: PLoS Comput Biol. 2009 Nov 13;5(11):e1000562. doi: 10.1371/journal.pcbi.1000562 (PMC2770120; doi:10.1371/journal.pcbi.1000562)
Supplement: Text S7 — ChIP-chip data sets (0.04 MB PDF) [file pcbi.1000562.s015.pdf]

Supplementary Text S7: ChIP-chip data sets

We also ran the methods on a collection contained four sets of real data from human or mouse: three ChIP-chip'ed TFs from retinioc acid-stimulated HL-60 cells from the Affymetrix set within the ENCODE project [4] corresponding to 1% of the human genome, and a whole genome ChIP-chip aimed to find TFBS for the ESR1 in mouse liver cells [3].

ChIP-chip has low resolution, and generally gives 0.5-1kb fragments in which the site is hidden. The correct binding sites within the larger regions have not been mapped, so the evaluation cannot depend on predicted positions of sites. There are also cases where chip regions do not have any reasonable consensus sites for the factor in question, either due to methodological noise or indirect binding. Therefore, instead of comparing to site locations, we aligned the output matrix from the program in question to all known JASPAR matrices using two different alignment methods: a modified Needleman-Wunsch algorithm [1] or an ungapped Smith-Waterman[2]. In the former an alignment of two matrices gives a score between 0 and 2 times the width of the smallest of the two matrices, which is translated into a percentage of the maximum score. In the latter an E-value is provided. Several motifs are very similar (i.e. nuclear receptors) so we recorded the rank of any correct motif that were among the 10 best scoring (Table S7). While it is clear that the ENCODE sets are more challenging than the ESR1 set for all methods, in any combined set and alignment method where one of the methods has the correct motif ranked among the 10 best, MoAn has the highest rank, with one exception- a shared rank of 1 between NestedMICA and MoAn on the ESR1 set using the Needleman-Wunsch alignment. By visual inspection of the logos of the resulting patterns it seems that the other methods are prone to find low-complexity AT-rich sequences that do not resemble TFBSs in the ENCODE sets (Table S7). Due to the few sets and relatively small difference between methods this was not included in the main article.

For the two methods that provide an output matrix (MoAn and NestedMICA) we plotted a ROC curve (Fig. S3) for the ESR1 set showing the discriminatory power of the matrix with respect to the positive and negative sets as a function of cutoff. This uses the standard mouse negative set as the false positives and the ESR1 set as the true positives. The matrix produced by MoAn is clearly more discriminatory than NestedMICAs.

The reason for not simply selecting all ENCODE chip-chip sets for evaluation is that the regions reported are not comparable between groups - some reported chip regions are pre-processed so that they have unreasonably short lengths, while others show consecutive regions (with no interval between them) as separate entities with different binding strength. We choose the Affymetrix subset ("Affy Sites"), downloaded from the UCSC browser, as in this study the authors have applied a sensible normalization using triplicates, and then detected regions that are enriched, merging consecutive enriched probes. We focused on the transcription factors RARA, CEBPe and PU1 as these are the TFs in the set for which we have a reasonable idea what they bind to (much of the remaining tracks are targeting modified histones, or the initiation site complex). The ESR1 set is taken directly from the cited study, which used similar methodology. The sizes of the sets and what is used as a negative set is listed in table S6.

References

[1] Sandelin, A. and Hoglund, A. and Lenhard, B. and Wasserman, W. W. (2003) Integrated analysis of yeast regulatory sequences for biologically linked clusters of genes *Funct Integr Genomics*, 3 (3) 125-134

[2] Mahony, S. and Benos, P.V. (2007) STAMP: a web tool for exploring DNA-binding motif similarities *Nucleic Acids Research*

[3] Gao, H. et al. Genome-wide identification of estrogen receptor alpha-binding sites in mouse liver *Mol Endocrinol* 22 (1) p10-22

[4] Birney, E. et al. (2007) Identification and analysis of functional elements in 1% of the human genome by the ENCODE pilot project *Nature* 447 p799-816
