# Supplementary material for: Discovery of Regulatory Elements is Improved by a Discriminatory Approach
Source: PLoS Comput Biol. 2009 Nov 13;5(11):e1000562. doi: 10.1371/journal.pcbi.1000562 (PMC2770120; doi:10.1371/journal.pcbi.1000562)
Supplement: Table S1 — Length of upstream and downstream extensions (0.01 MB PDF) [file pcbi.1000562.s016.pdf]

**Length**	**Upstream**	**Downstream**
200	150	50
300	200	100
400	300	100
600	500	100
800	650	150
1000	800	200
1200	1000	200
