# Supplementary figures and images for: Discovery of Regulatory Elements is Improved by a Discriminatory Approach
Source: PLoS Comput Biol. 2009 Nov 13;5(11):e1000562. doi: 10.1371/journal.pcbi.1000562 (PMC2770120; doi:10.1371/journal.pcbi.1000562)

| TF        | JASPAR                                                                            | TF    | JASPAR                                                                              |
|-----------|-----------------------------------------------------------------------------------|-------|-------------------------------------------------------------------------------------|
| Arnt      | 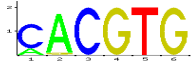 | CREB1 | 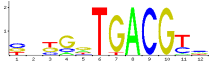 |
| ELK1      | 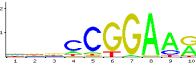 | Evi1  | 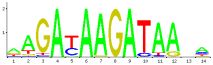 |
| FOXD1     | 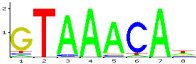 | HLF   | 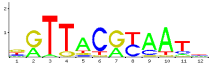 |
| IRF2      | 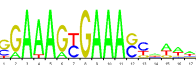 | MEF2A | 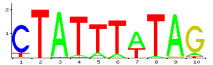 |
| NF-kappaB | 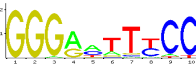 | NR2F1 | 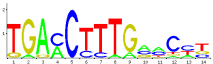 |
| SOX9      | 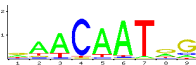 | TCF1  | 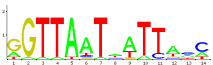 |

Supplement: Table S2 — Motifs planted in single occurrence sets (0.04 MB PDF) [file pcbi.1000562.s017.pdf]

| TF 1     | JASPAR                                                                            | TF 2   | JASPAR                                                                              |
|----------|-----------------------------------------------------------------------------------|--------|-------------------------------------------------------------------------------------|
| Arnt-Ahr | 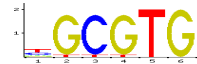 | Nkx2-5 | 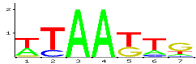 |
| T        | 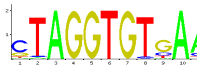 | Mycn   | 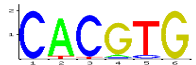 |
| Pax5     | 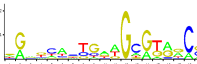 | GATA3  | 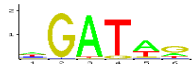 |
| En1      | 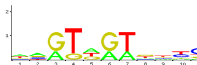 | Sox17  | 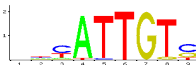 |
| Prrx2    | 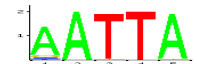 | Bapx1  | 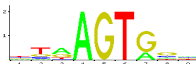 |

Supplement: Table S3 — Motifs planted in co-occurrence sets (0.03 MB PDF) [file pcbi.1000562.s018.pdf]
