# Supplementary material for: Discovery of Regulatory Elements is Improved by a Discriminatory Approach
Source: PLoS Comput Biol. 2009 Nov 13;5(11):e1000562. doi: 10.1371/journal.pcbi.1000562 (PMC2770120; doi:10.1371/journal.pcbi.1000562)
Supplement: Table S4 — Results on the mammalian subset of the Tompa assessment (0.01 MB PDF) [file pcbi.1000562.s019.pdf]

Method	nPPV	nSn	nSp	nCC	nPC	sPPV	sSn	sASP
AlignACE	0.091	0.026	0.995	0.039	0.021	0.093	0.042	0.067
ANN-Spec	0.089	0.074	0.985	0.064	0.042	0.086	0.136	0.111
Consensus	0.027	0.002	0.998	0.002	0.002	0.043	0.007	0.025
GLAM	0.038	0.022	0.988	0.014	0.014	0.058	0.038	0.048
Improbizer	0.076	0.061	0.985	0.051	0.035	0.083	0.112	0.097
MEME	0.079	0.047	0.989	0.046	0.030	0.103	0.073	0.088
MEME3	0.064	0.065	0.981	0.045	0.033	0.099	0.101	0.100
MITRA	0.050	0.023	0.991	0.020	0.016	0.060	0.045	0.053
MoAn	0.117	0.053	0.992	0.066	0.038	0.141	0.080	0.111
MotifSampler	0.054	0.030	0.990	0.026	0.020	0.064	0.059	0.062
oligodyad	0.227	0.025	0.998	0.069	0.023	0.189	0.049	0.119
QuickScore	0.038	0.016	0.992	0.012	0.011	0.030	0.035	0.032
SeSiMCMC	0.029	0.058	0.961	0.014	0.020	0.045	0.052	0.049
Weeder	0.144	0.033	0.996	0.060	0.027	0.136	0.063	0.100
YMF	0.090	0.047	0.990	0.051	0.032	0.071	0.077	0.074
