# Supplementary material for: Discovery of Regulatory Elements is Improved by a Discriminatory Approach
Source: PLoS Comput Biol. 2009 Nov 13;5(11):e1000562. doi: 10.1371/journal.pcbi.1000562 (PMC2770120; doi:10.1371/journal.pcbi.1000562)
Supplement: Table S5 — Sizes of PAZAR data sets (0.01 MB PDF) [file pcbi.1000562.s020.pdf]

**Set**	**Size**	**Negative set**
ARE *Mus musculus*	14	1000 mouse promoters (-1000-+200)
ARE *Homo sapiens*	24	1000 human promoters (-1000-+200)
HNF4 *Mus musculus*	74	1000 mouse promoters (-1000-+200)
HNF4 *Homo sapiens*	84	1000 human promoters (-1000-+200)
RARE *Mus musculus*	76	1000 mouse promoters (-1000-+200)
HNF4 *Homo sapiens*	118	1000 human promoters (-1000-+200)
STAT1 *Homo sapiens*	56	1000 human promoters (-1000-+200)
