# Supplementary material for: Discovery of Regulatory Elements is Improved by a Discriminatory Approach
Source: PLoS Comput Biol. 2009 Nov 13;5(11):e1000562. doi: 10.1371/journal.pcbi.1000562 (PMC2770120; doi:10.1371/journal.pcbi.1000562)
Supplement: Table S6 — Sizes of ENCODE data sets (0.01 MB PDF) [file pcbi.1000562.s021.pdf]

**Set**	**Size**	**Negative set**
ESR1	948	1000 mouse promoters (-1000-+200)
Cebp	1351	1000 human promoters (-1000-+200)
Pu1	565	1000 human promoters (-1000-+200)
Rara	1672	1000 human promoters (-1000-+200)
