# Supplementary material for: Discovery of Regulatory Elements is Improved by a Discriminatory Approach
Source: PLoS Comput Biol. 2009 Nov 13;5(11):e1000562. doi: 10.1371/journal.pcbi.1000562 (PMC2770120; doi:10.1371/journal.pcbi.1000562)
Supplement: Table S7 — Performance on ENCODE data sets (0.07 MB PDF) [file pcbi.1000562.s022.pdf]

(a) MoAn

Motifs	Predicted logo	Jaspar logo	Jaspar rank	STAMP rank
ESR1	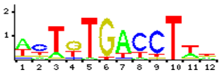	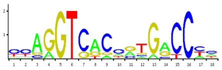	1	2
Cebp	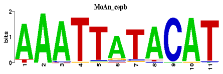	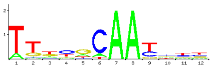	7	-
Pu1	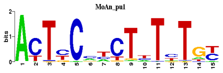	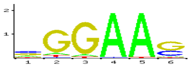	-	4
Rara	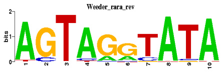	AGGTCA	NA	NA

(b) DEME

Motifs	Predicted logo	Jaspar logo	Jaspar rank	STAMP rank
ESR1	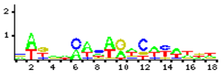	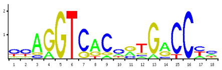	2	7
Cebp	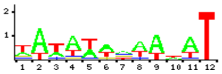	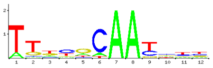	-	-
Pu1	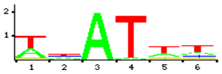	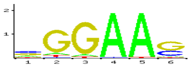	-	-
Rara	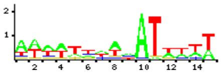	AGGTCA	NA	NA

(c) MEME

Motifs	Predicted logo	Jaspar logo	Jaspar rank	STAMP rank
ESR1	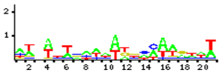	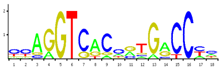	-	-
Cebp	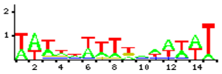	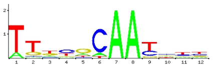	-	-
Pu1	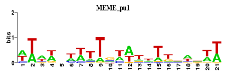	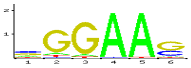	-	-
Rara	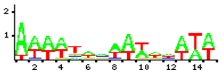	AGGTCA	NA	NA

(d) Weeder

Motifs	Predicted logo	Jaspar logo	Jaspar rank	STAMP rank
ESR1	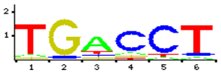	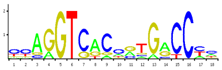	6	-
Cebp	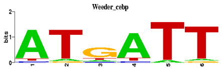	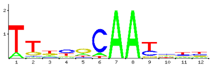	-	-
Pu1	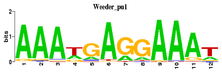	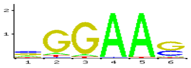	-	-
Rara	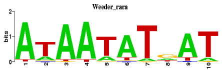	AGGTCA	NA	NA

(e) NestedMICA

Motifs	Predicted logo	Jaspar logo	Jaspar rank	STAMP rank
ESR1	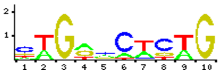	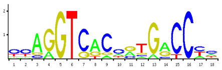	1	-
Cebp	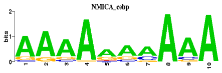	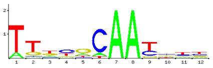	-	-
Pu1	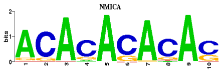	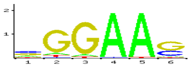	-	-
Rara	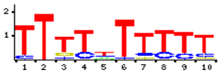	AGGTCA	NA	NA
